# Massively parallel sequencing and capillary electrophoresis of a novel panel of falcon STRs: Concordance with minisatellite DNA profiles from historical wildlife crime

**DOI:** 10.1016/j.fsigen.2021.102550

**Published:** 2021-09

**Authors:** Jordan Beasley, Guy Shorrock, Rita Neumann, Celia A. May, Jon H. Wetton

**Affiliations:** a Department of Genetics & Genome Biology, University of Leicester, Leicester LE1 7RH, UK; b RSPB Investigations Team, The Lodge, Potton Road, Sandy, Bedfordshire SG19 2DL, UK

**Keywords:** DNA forensics, STR multiplex, Massively parallel sequencing, Wildlife laundering, Peregrine, Raptor persecution

## Abstract

Birds of prey have suffered persecution for centuries through trapping, shooting, poisoning and theft from the wild to meet the demand from egg collectors and falconers; they were also amongst the earliest beneficiaries of DNA testing in wildlife forensics. Here we report the identification and characterisation of 14 novel tetramer, pentamer and hexamer short tandem repeat (STR) markers which can be typed either by capillary electrophoresis or massively parallel sequencing (MPS) and apply them to historical casework samples involving 49 peregrine falcons, 30 of which were claimed to be the captively bred offspring of nine pairs. The birds were initially tested in 1994 with a multilocus DNA fingerprinting probe, a sex test and eight single-locus minisatellite probes (SLPs) demonstrating that 23 birds were unrelated to the claimed parents. The multilocus and SLP approaches were highly discriminating but extremely time consuming and required microgram quantities of high molecular weight DNA and the use of radioisotopes. The STR markers displayed between 2 and 21 alleles per locus (mean = 7.6), lengths between 140 and 360 bp, and heterozygosities from 0.4 to 0.93. They produced wholly concordant conclusions with similar discrimination power but in a fraction of the time using a hundred-fold less DNA and with standard forensic equipment. Furthermore, eleven of these STRs were amplified in a single reaction and typed using MPS on the Illumina MiSeq platform revealing eight additional alleles (three with variant repeat structures and five solely due to flanking SNPs) across four loci. This approach gave a random match probability of < 1E-9, and a parental pair false inclusion probability of < 1E-5, with a further ten-fold reduction in the amount of DNA required (~3 ng) and the potential to analyse mixed samples. These STRs will be of value in monitoring wild populations of these key indicator species as well as for testing captive breeding claims and establishing a database of captive raptors. They have the potential to resolve complex cases involving trace, mixed and degraded samples from raptor persecution casework representing a significant advance over the previously applied methods.

## Highlights

• Mini- and microsatellite DNA profiles identify false falcon captive breeding claims.
• 12 novel tetra- to hexamer STRs used in capillary electrophoresis multiplexes.
• First application of massively parallel sequencing to a wildlife crime case.
• Massively parallel sequencing enhances discrimination by STR motif and SNP variants.
• Impact of DNA testing on raptor wildlife crime and conservation.

## Graphical Abstract

**Figure:**
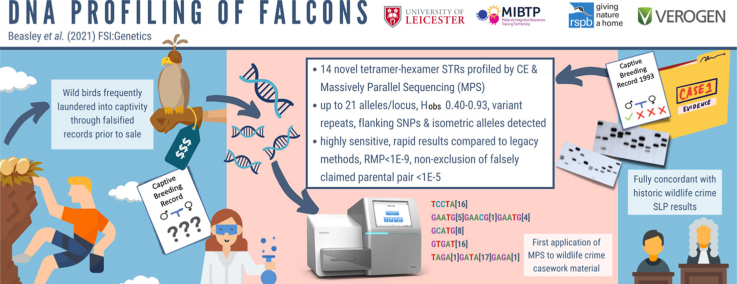

## 1 Introduction

Birds of prey, like many predatory species, have been subject to human persecution for centuries and this continues to be a global concern [28]. In the United Kingdom persecution has taken many forms including shooting, trapping, and poisoning by landowners and gamekeepers who consider them a threat to livestock and gamebirds [34]. In addition, many were illegally taken from the wild as eggs or nestlings and ‘laundered’ into the captive-bred population to meet the national and international demand for falconry birds [13], [50]. The Wildlife and Countryside Act 1981 introduced a Bird Registration Scheme (BRS) for certain rare birds held in captivity in Great Britain. This was intended to make keepers accountable for birds in their possession and to prevent wild-taken birds being laundered into the captive market. The scheme required declaration of the identity of the parent birds when captive-bred nestlings were fitted with a uniquely numbered permanent ring within the first month of life [13]. For people with experience in the keeping and breeding of certain species, the BRS was relatively simple to bypass. Species like peregrine falcon (*Falco peregrinus*) and goshawk (*Accipiter gentilis*), were highly sought after for falconry, difficult to breed in captivity, and commanded high prices. By obtaining wild-taken eggs or young chicks the birds could be declared as captive bred, registered and effectively legitimised, then sold. The fact that some keepers involved were also successfully breeding these birds in captivity and used wild stock to boost claimed productivity provided a further cover for illegal activities. However, there was little that could be done to check the legitimacy of claimed relationships prior to the advent of DNA testing.

In 1991, the Royal Society for the Protection of Birds (RSPB) initiated the first ever use of DNA testing in the UK to investigate a wildlife crime relating to the alleged captive breeding of goshawks. A human-derived DNA fingerprinting probe indicated four sibling chicks were highly unlikely to be related to the putative female parent. The accused later pleaded guilty to unlawful possession of the chicks; the first wildlife conviction based on DNA testing [36]. Whilst revolutionary, DNA fingerprinting was poorly suited to large-scale applications as the complex multi-locus profiles comprising alleles from tens of loci were difficult to compare reliably [19], [20]. This led to the development of single-locus profiling (SLP) in which a succession of radioisotope-labelled probes was used to detect the alleles at a number of defined minisatellite loci [53]. This approach required the cloning of a panel of minisatellites from birds of prey so that the homologous locus alone would be detected [49]. Between 1993 and 1997 SLP probes were used in cases involving more than 350 birds of prey resulting in twelve convictions for a variety of offences involving the laundering of wild birds. The SLP technique required specialist facilities for handling radioisotopes and was extremely labour-intensive, leading to its rapid replacement in human forensics by capillary electrophoretic (CE) screening of panels of microsatellites, also known as short tandem repeats (STRs).

Recent advances in DNA sequencing technology have begun to impact the field of human DNA forensics. In particular, the use of massively parallel sequencing (MPS) in place of CE for the analysis of STRs opens up further advantages [11]. Since MPS is not reliant on discrimination by length, there is greater multiplexing capability (e.g. validated human forensic MPS kits can simultaneously interrogate 27 autosomal, 24 Y-chromosomal and 7 X-chromosomal STRs, along with 172 SNPs [18]). This multi-target approach in a single reaction further conserves DNA which may be very limited in forensic cases, and as the method is not reliant on differentiating both alleles and loci by length shorter amplicons with overlapping size ranges can be analysed which benefits the analysis of degraded DNA. By establishing the sequences of STR alleles rather than simply determining their overall length, MPS exploits all the existing variation, for example allowing discrimination between isometric alleles [17], [54] and can thus reduce random match probabilities. Additionally, MPS has the potential to provide more accurate information on mixed DNA samples – a stutter from a major contributor and a real allele from a minor contributor could appear as one peak in CE analysis but they may be distinguishable by MPS [31].

In this study we describe the identification and characterisation of a panel of novel tetra- to hexanucleotide repeat STRs for falcons to replace the dinucleotide markers widely used in falcon population studies (e.g. [9]) which are now considered unsuitable for UK casework. We demonstrate how they can be implemented using either conventional fluorescent multiplexing and CE, or MPS approaches, by applying them to historical casework samples previously typed with SLP markers. The STR markers provide fully concordant results with equivalent statistical power but with much greater sensitivity, ease of use and potential for application in a wider range of investigations. We highlight the advantages of each approach and provide the first example of how MPS could be used in the investigation of wildlife crime.

## 2 Materials and Methods

### 2.1 DNA sampling

Samples were obtained from 49 peregrines as part of an investigation into a falconer’s captive breeding claims submitted to the registration scheme for protected species (BRS) since 1987, following a dramatic increase in claimed breeding success in 1993 [44]. The falconer had retained most of the claimed parents but had sold many of the 30 allegedly captive-bred young to innocent purchasers. During simultaneous raids conducted by nine English police forces in February 1994, qualified veterinarians collected blood by brachial venipuncture into EDTA tubes from all relevant birds.

### 2.2 Bioinformatic selection of STRs

The novel STR loci were identified from the genome of a wild-caught male peregrine sequenced to a coverage of 137x and published as part of the Avian Phylogenetic Project [55] [GenBank accessions GCA_000337955.1 (7021 unplaced scaffolds, N50 = 3,935,757) and GCA_001887755.1 (72 chromosome-assigned scaffolds, N50 = 26,776,924)]. A variety of *in silico* tools were used to maximise discovery of informative markers and to retrieve perfect, compound, and interrupted repeat arrays. RepeatMasker [39], MISA [3], GMATA [46] and MSATCOMMANDER [12] were chosen both for their ability to handle large input files and the ease with which flanking regions could be extracted for primer design. Search criteria were set to identify repeat motifs of 4–6 bp in length and for the repeat number to range between 8 and 16 copies.

Primers were designed using Primer 3 [41] for a subset of 24 from the > 7500 retrieved loci representing a selection of repeat motifs. The product size range was set to 100–400 bp in order to be compatible with MPS. Primer pairs were initially tested by CE in a small screening panel to assess variability within and between four falcon species (peregrine *F. peregrinus*, merlin *F. columbarius*, gyrfalcon *F. rusticolus* and saker *F. cherrug*) and to determine empirically annealing temperature range. A set of 14 of these STRs was subsequently used in multiplex reactions. These newly reported STR loci were named as follows: *Fpe*μN_N e.g. where *Fpe*μ435_1 = *Falco peregrinus* microsatellite within peregrine genome scaffold 435_1.

### 2.3 DNA amplification and fragment detection

The 14 STRs from the bioinformatic pipeline were chosen to amplify under the same conditions. Multiplex-PCR reactions were set up according to Table S1 using the Type-It Microsatellite PCR kit (Qiagen) with 1–5 ng of input DNA per reaction. Amplification was performed in Veriti PCR machines (Applied Biosystems) for 27 cycles, denaturing at 95 °C for 30 s, annealing for 90 s at 60 °C and extending for 30 s at 72 °C, followed by a final incubation of 30 min at 60 °C. In addition, two “legacy” STRs *Fpe*μ1 [GATG]_n_ and *Fpe*μ2 [GGAAGA]_n_ cloned by one of the authors in 1994 (see Supplementary Text) were used for CE typing as they met the desired criteria of low stutter, high allele diversity and heterozygosity. The two cloned STRs required different conditions – an initial incubation at 94 °C for 3 min, then 30 cycles of: 94 °C for 45 s, annealing at 58/55 °C for 1 min (*Fpe*μ1 and *Fpe*μ2 respectively), 68 °C for 90 s followed by a final extension at 72 °C for 5 min using BIOTAQ DNA polymerase and associated 10x buffer with 50 mM MgCl_2_ (Bioline). Aliquots of 1 μl of amplification product were each mixed with 8.5 μl of Hi-Di formamide and 0.5 μl of GeneScan™ 600 LIZ® Size Standard (Applied Biosystems), heated for 5 min at 95 °C and then immediately cooled to 4 °C. The denatured PCR products were then run on an ABI PRISM® 3130xl Genetic Analyzer (Applied Biosystems) using POP-7 polymer on a 36 cm array and the resulting profiles were examined using GeneMapper software (version 4.0).

### 2.4 Sanger sequencing of legacy STRs

In order to establish an appropriate allele nomenclature and determine the basis of intermediate allele lengths, several *Fpe*μ1 and *Fpe*μ2 alleles were PCR-amplified and then purified using the Zymoclean Gel DNA recovery kit prior to Sanger-sequencing using Applied Biosystems BigDye Terminator v3.1 chemistry according to manufacturers’ instructions. The *Fpe*µ1 templates were prepared and sequenced using the primers listed in Table S1 whilst the following pair of primers was used to amplify and sequence *Fpe*µ2 templates: Fu2_F2 (5′ ATATGGCACGGCACAGCTCA-3′) plus Fu2_R3 (5′ GTCCTTGAATGCTTCCAGAG-3′).

### 2.5 Massively parallel sequencing of STRs

The bioinformatically-identified STRs were amplified in a single multiplex using 3–10 ng of genomic DNA per bird with each unlabelled primer at a final concentration of 0.2 μM. Barcoded sequencing libraries were prepared with the TruSeq PCR-free Library Preparation kit (Illumina) and quantified using both the 2100 BioAnalyser system (Agilent) and Qubit™ fluorometer (Invitrogen) with appropriate dsDNA high sensitivity assays. Based on a 550 bp average insert size, libraries were normalised to 4 nM, diluted, and denatured to a loading concentration of 12 pM with a 1% ΦX spike-in in accordance with the manufacturer’s recommendations. Paired-end sequencing was performed using 500 cycles on an Illumina/Verogen MiSeq FGx sequencer in “research use only” (RUO) mode using the machine’s “Generate FASTQ” workflow. Paired-end reads were merged with FLASH version 1.2.11/lo and alleles were called by FDSTools ([56]).

### 2.6 Forensic and statistical analysis

The software package ML-Relate [24] was used to screen for any undeclared relatives within the dataset. STRAF 1.0.5 [15] was used to calculate allele frequencies, polymorphic information content (PIC), expected and observed heterozygosity (H_exp_, H_obs_), test for deviation from Hardy-Weinberg equilibrium and perform LD analysis. Combined non-exclusion probabilities for a single parent (NE-1P), a parent pair (NE-PP), identity (NE-I) and sib-identity (NE-SI) were calculated with Cervus 3.0.7 [25], [29], [38].

## 3 Results

Sixteen STRs were amplified in all individuals, two identified by cloning in the 1990s plus 14 selected bioinformatically in 2017 from the published peregrine genome. The latter set of markers were chosen for their collective potential to discriminate between individuals in small panels of different falcon species and of these, twelve proved to be easily and robustly typed in this larger sample of peregrines. Two loci were excluded from further analysis: *Fpe*μ98_2 due to poor amplification and *Fpe*μ25_1 due to apparent detection of more than one locus in peregrines under the shared multiplex PCR conditions. In total, therefore, data from 14 STRs were interpreted in this study (*Fpe*μ1, *Fpe*μ2, *Fpe*μ12_1, *Fpe*μ145_1, *Fpe*μ208_1, *Fpe*μ248_1, *Fpe*μ26_1, *Fpe*μ298_1, *Fpe*μ33_1, *Fpe*μ342_1, *Fpe*μ353_1, *Fpe*μ435_1, *Fpe*μ46_1, *Fpe*μ56_1). The observed amplicon size ranges of these STR loci span 140–360 bp as shown in Fig. S1. The potential size overlap of at least nine loci between 240 and 250 bp necessitated analysis of multiplexes in separate CE capillaries (Table S1), although co-amplification of these loci was possible with MPS. Nine minisatellite SLP markers typed for all 49 birds in 1994 (see Supplementary Text) were used to provide concordance data, select unrelated birds for STR allele population frequency estimation and to identify genuine parent/offspring groups for exploring possible linkage at the STR loci typed by CE and MPS in 2020.

### 3.1 Sex-linked markers

The sexes of 25 adult birds (14 males and 11 females) were known from their registration documents. The c*Mmi*12 minisatellite probe, which has previously been shown to detect a W-linked locus in female peregrine falcons [48], detected one of two W-chromosome-linked alleles (α or β) in all adult females and none of the adult males (P < 3.0E-08, binomial). This probe was used only to assign the sexes of the remaining 24 birds (11 males and 13 females), which included 15 of 18 juveniles from 1993. The STR locus *Fpe*μ435_1 which lies within an unmapped scaffold of the peregrine reference genome displayed apparent Z-chromosome linkage being heterozygous in 16 of the 25 males and none of the 24 females (P = 0.0002, Fisher’s Exact Test). BLAST searching of this locus has shown it to lie within the Z-chromosome (NC_054080.1) of the recently released lesser kestrel (*Falco naumanni*) genome (GCA_017639655.1); all other markers appeared to be autosomal.

### 3.2 Mendelian inheritance and parentage

**Fig. 1:**
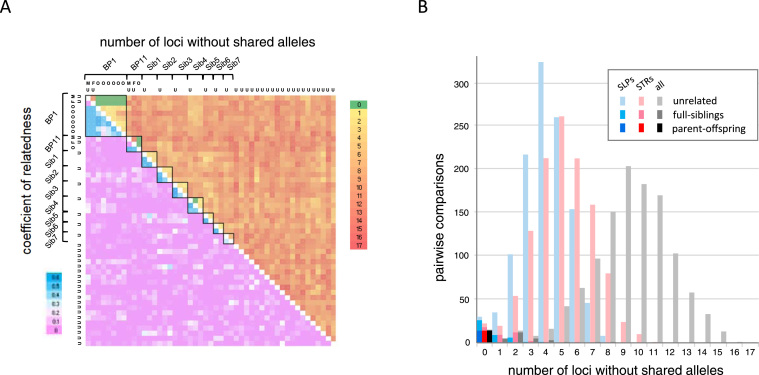
Determining relatedness using all 22 SLP and STR markers. (A) Figure shows pairwise comparisons between each of the 49 peregrines. Above the diagonal, increasing numbers of loci at which no alleles are shared are indicated on a scale from 0 to 22 (green to red). Below the diagonal are the coefficients of relatedness (r) as determined by ML-Relate; likely first-degree relatives are shown by blue shades, these are clustered along the diagonal and grouped by heavy black boxes. The first two boxes from left to right (and top to bottom) correspond to the adults and confirmed offspring of breeding pair BP1 (two adults (M,F), six offspring (O)) and BP11 (two adults, one offspring) with at least one shared allele at every locus. The following seven boxes enclose inferred full sibships (Sib1–7). The remaining 20 individuals appear unrelated to all others. The 31 individuals marked (U) were used to estimate allele frequencies. (B) The number of loci showing no shared alleles between confirmed parent/offspring (dark shades), inferred full sibling (mid shades) and more distant relationships (light shades) are shown in the histograms for the eight minisatellite SLPs (blue), 14 CE STRs (red) and all markers combined (grey). (For interpretation of the references to colour in this figure legend, the reader is referred to the web version of this article.)

**Fig. 2:**
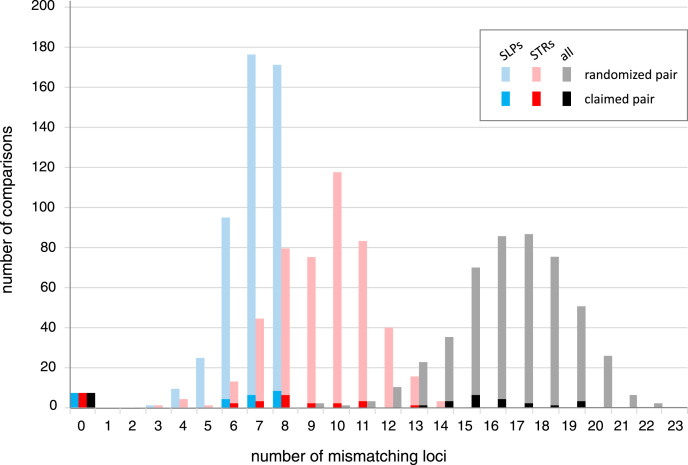
Genetic incompatibilities between young and claimed breeding pairs. Number of mismatching loci in pairwise comparisons between the young birds and both claimed (dark shades) and all other breeding pairs (light shades) with eight minisatellite SLPs (blue), 14 CE STRs (red) and all markers combined (grey). (For interpretation of the references to colour in this figure legend, the reader is referred to the web version of this article.)

Registration records were used to test the declared relationships between the birds. The thirty alleged “offspring” were the claimed progeny of nine of the eleven “breeding” pairs the accused possessed at various times during the seven years covered by the investigation. We determined the number of loci that displayed compatible genotypes with the recorded pair, individual claimed parents, and all adult birds with both minisatellite and STR markers. Pairwise comparison of all 49 individuals to determine the number of loci at which no alleles were shared and a similar analysis in which the number of loci that are incompatible with a claimed breeding pair/offspring trio are shown in Figs. 1 and 2 respectively. The full suite of 22 tested markers was analysed with ML-Relate to estimate the coefficients of relatedness (r) between all 49 birds and this aided categorisation into true first-degree relatives (parent/offspring and putative full-sibs) and probable unrelated individuals which could be used to produce an unbiased estimation of allele frequency (Fig. 1).

Twenty-three young, including 16 of 18 birds declared as captively bred in 1993, were clearly excluded as being the offspring of any pair that the accused possessed with at least 13 loci incompatible with the declared parents, and mismatches at no fewer than 8 loci with all other adult pairs. Seven birds were shown to be wholly compatible with their claimed parents: six were the offspring of breeding pair 1 (BP1, one each in 1987, 1989, 1992 and 1993, and two in 1990) and one was the offspring of breeding pair 11 (BP11 in 1993). BP11 comprised a female peregrine (*Falco p. peregrinus*) paired with a male Barbary Falcon (*Falco p. pelegrinoides*), a sub-species that shows very strong assortative mating where it overlaps with other peregrine subspecies in the wild and was originally classified by some authors as a separate species ([52] *c.f.* [43]).

The inheritance of a null allele was deduced at *Fpe*μ12_1 in the legitimate family of BP1 which showed no incompatibilities other than an absence of the adult female’s single detectable allele in four of her seemingly homozygous offspring. Primers were designed outside of the original amplicon to allow Sanger sequencing of the original primer binding sites and this identified a single base insertion at the 3’ end of the reverse primer binding site. A redesigned primer (5′-GCATTGCATCTATTCTGTTCC**A**G-3′) including the insertion successfully amplified the null as a 14-repeat allele and confirmed the legitimacy of this family. The pattern of parental inclusion and exclusion observed with the STR markers was wholly concordant with the previous SLP typing results as reported in court.

Among the young excluded as offspring of the captive adults, seven potential full-sibling groups (Sib1–7 in Fig. 1) could be identified based on their high coefficients of relatedness (r > 0.33), few loci without shared alleles (mean = 2.4, range 0–7), and possession of a maximum of four alleles at any one locus. Subsequent checks revealed that the putative siblings within each of the groups Sib 1–5 had hatched within a day of each other implying that they came from the same clutch of eggs, whereas the two birds grouped in Sib 6 were born two years apart. Sib 7 comprised two birds for which no registration documents were available.

A single individual was chosen at random from each of the groups (Sib1–7), and along with the four adults from BP1 and BP11, and 20 remaining apparently unrelated individuals this provided 15 males and 16 females which were used to establish allele frequencies and standard forensic statistics for the contemporaneous UK captive population (see Table 1).

**Table 1:** CE STR and minisatellite SLP allele frequencies among the 31 unrelated birds STRs are denoted by the number of repeats (as determined by Sanger sequencing and/or MPS see Tables S4 & S5); allele 14.n at *Fpe*µ12_1 corresponds to the null rescued with an alternative primer. Due to the larger number of repeats at *Fpe*µ2 the corresponding allele designation for this locus is provided in the adjacent column. Minisatellite alleles are grouped into sequential distinguishable bins based upon their occurrence among all 49 birds, those marked (*) were not present in the 31 randomly selected unrelated birds as determined by ML-Relate but were observed in their putative siblings. N = number of alleles analysed; as *Fpe*μ435_1 is Z-linked there were 46 alleles among the 15 males and 16 females. Nall = number of alleles (distinguishable by CE alone for STRs), expected and observed heterozygosity (H_exp_ and H_obs_), pHW = probability of Hardy-Weinberg equilibrium (prior to Bonferroni correction). NE-1P = probability that a locus will not exclude an unrelated candidate parent from parentage when the genotype of the other parent is unknown. NE-PP = corresponding value for an unrelated pair where both genotypes are known. NE-I = probability that unrelated individuals share a genotype and NE-SI = corresponding value for siblings.

Microsatellite (STR)																Minisatellite (SLP)								
Allele	*Fpe*µ1	*Fpe*µ298_1	*Fpe*µ46_2	*Fpe*µ342_1	*Fpe*µ435_1	*Fpe*µ208_1	*Fpe*µ248_1	*Fpe*µ26_1	*Fpe*µ145_1	*Fpe*µ353_1	*Fpe*µ12_1	*Fpe*µ33_1	*Fpe*µ56_1	*Fpe*µ2	Allele	Allele	c*Fpe*MS13	c*Fpe*MS1	c*Fpe*MS15	c*Fco*MS2	c*Fti*1	c*Fpe*MS17	c*Fpe*MS5	c*Fco*MS19
7										0.032				0.016	21.5	A	0.016	0.210	0.032	0.048	0.226	0.016	0.016	0.048
8										0.065				0.016	23	B	0.097	0.081	0.274	0.016	0.113	0.194	0.129	0.097
9										0.435				0.048	24	C	*	0.194	0.097	0.306	0.016	0.016	0.032	0.113
10		0.097			0.0652	0.097		0.339	0.097	0.274	0.145			0.032	27	D	0.177	0.016	0.048	0.081	0.081	0.016	0.210	0.226
11						0.194	0.016	0.355	0.226		0.016	0.081		0.081	27.5	E	0.016	0.032	0.048	0.129	0.032	0.016	0.113	0.194
11.1				0.016										0.194	29	F	0.016	0.274	0.065	0.290	*	0.113	0.081	0.065
12		0.339	0.371			0.081			0.032		0.258	0.032		0.016	30	G	0.065	0.016	0.145	0.065	0.032	0.210	0.161	0.145
13		0.065	0.629	0.032		0.548			0.274		0.097	0.484		0.016	32	H	0.065	0.097	0.161	0.065	0.032	0.210	0.065	0.081
14	0.065	0.500		0.016					0.065		0.194	0.097		0.016	32.5	I	0.242	0.032	0.048		0.032	0.210	0.194	0.032
14.n											0.016			0.016	33	J	0.016	0.048	0.048		0.387			
14.1				0.355										0.016	33.5	K	0.016		0.016		0.048			
14.3										0.194				0.048	34	L	0.048		0.016					
15	0.290			0.016	0.3043	0.016			0.145		0.194	0.113	0.016	0.081	34.5	M	0.194							
15.1				0.016										0.065	35	N	0.032							
16	0.016			0.032	0.0217	0.065	0.226	0.016	0.048		0.048	0.129	0.065	0.016	35.5									
17	0.323			0.016	0.587		0.032	0.048	0.113		0.016	0.065		0.129	36									
18	0.177			0.468			0.016	0.016					0.403	0.048	37									
19	0.032						0.226	0.210			0.016		0.274	0.048	38.5									
20	0.081			0.032	0.0217		0.435	0.016					0.065	0.065	40.5									
21	0.016												0.177	0.016	44.5									
22							0.048							0.016	46									
N	62	62	62	62	46	62	62	62	62	62	62	62	62	62		N	62	62	62	62	62	62	62	62
Nall	8	4	2	10	5	6	7	7	8	5	10	7	6	21		Nall	13	10	12	8	10	9	9	9
PIC	0.733	0.554	0.358	0.593	0.445	0.605	0.658	0.660	0.801	0.641	0.802	0.690	0.678	0.906		PIC	0.834	0.800	0.839	0.759	0.747	0.791	0.834	0.838
PM	0.138	0.203	0.378	0.182	0.440	0.176	0.153	0.147	0.074	0.190	0.070	0.124	0.138	0.041		PM	0.061	0.080	0.076	0.109	0.101	0.076	0.070	0.072
PE	0.673	0.174	0.126	0.268	0.482	0.149	0.174	0.394	0.552	0.611	0.349	0.307	0.394	0.868		PE	0.673	0.349	0.868	0.611	0.349	0.673	0.737	0.868
NE-1P	0.620	0.794	0.891	0.755	0.731	0.757	0.708	0.707	0.520	0.727	0.520	0.668	0.687	0.298		NE-1P	0.458	0.519	0.446	0.582	0.593	0.542	0.464	0.454
NE-PP	0.255	0.482	0.729	0.415	0.387	0.382	0.350	0.360	0.169	0.379	0.170	0.273	0.327	0.048		NE-PP	0.126	0.168	0.116	0.216	0.218	0.190	0.132	0.125
NE-I	0.089	0.210	0.393	0.180	0.149	0.165	0.133	0.135	0.054	0.146	0.054	0.107	0.121	0.014		NE-I	0.039	0.054	0.036	0.074	0.078	0.060	0.040	0.038
NE-SI	0.388	0.492	0.615	0.470	0.442	0.470	0.431	0.428	0.352	0.440	0.351	0.419	0.419	0.298		NE-SI	0.335	0.352	0.332	0.375	0.383	0.357	0.334	0.332
Hexp	0.781	0.632	0.474	0.662	0.545	0.652	0.716	0.724	0.837	0.704	0.838	0.727	0.734	0.927		Hexp	0.864	0.836	0.867	0.801	0.786	0.830	0.865	0.868
Hobs	0.839	0.484	0.419	0.581	0.733	0.452	0.484	0.677	0.774	0.807	0.645	0.613	0.677	0.936		Hobs	0.839	0.645	0.936	0.807	0.645	0.839	0.871	0.936
pHW	0.101	0.113	0.715	0.219	0.185	0.099	0.004	0.321	0.202	0.242	0.038	0.171	0.446	0.589		pHW	0.594	0.010	0.132	0.260	0.056	0.808	0.264	0.325

### 3.3 Linkage

Physical locations of twelve of the STRs have been provisionally mapped to individual peregrine chromosomes [10], [23] see Table S2. Six of these map to the long arm of chromosome 4, including the two cloned STRs (*Fpe*μ1 and *Fpe*μ2) and four selected through the bioinformatic pipeline (*Fpe*μ33_1, *Fpe*μ56_1, *Fpe*μ298_1 and *Fpe*μ25_1, the last of which was not analysed in this study). Linkage between the analysed markers is detectable within the family group of BP1 comprising the two parents and their six offspring with the extent of coinheritance declining with increased physical distance, although the phasing of alleles cannot be confirmed with the two generations available. There is no evidence of recombination between *Fpe*μ2 (in scaffold 127_2) and *Fpe*μ56_1 which are approximately 16.1 Mb apart, and potentially only single recombinants between *Fpe*μ33_1 and *Fpe*μ2 (~10.5 Mb apart), and *Fpe*μ1 (in scaffold 9_1) and *Fpe*μ298_1 (~14.6 Mb apart). However, two recombinants are seen between *Fpe*μ56_1 and *Fpe*μ1 which are ~19.0 Mb apart, therefore at least four of the twelve informative meioses between the two most distal markers, *Fpe*μ33_1 and *Fpe*μ298_1 separated by ~60.2 Mb, are recombinant. Our data from more extensive pedigrees in other *Falco* species including some spanning three generations also show evidence of linkage between adjacent loci and free association between other markers on chromosome 4, reflecting a non-linear increase in recombination frequency with physical distance between markers, presumably due to intervening recombination hotspots and deserts [26], [37].

The coinheritance of linked alleles can result in heightened similarity but the inheritance of the alternate copies of linkage groups diminishes genetic similarity to a similar extent. Two pairwise comparisons between the confirmed offspring of BP1 which yielded very low estimates of r (0.21 and 0.17) and several loci without shared alleles (8 and 9, when comparing the second offspring in Fig. 1 with the fifth and sixth) result from the inheritance not only of the alternate copies of chromosome 4 from both parents but also of tightly linked minisatellites as discussed below.

In order to establish whether physical linkage leads to the maintenance of detectable associations between alleles among unrelated birds we explored levels of linkage disequilibrium (LD) among the sample of 31 unrelated birds representing the potential breeding stock of captive UK peregrines. There is no evidence of LD between any combination of STRs indicating that these markers can be considered independent at the population level [47] despite several being on the same chromosome. However, significant LD (P < 0.002 after Bonferroni correction) was observed between *Fpe*MS15 and *Fpe*MS17, the two most closely linked minisatellite loci [49]. This may reflect tighter physical linkage between these (as yet unmapped) markers but it is also possible that the high levels of allele diversity which result from the typical pattern of minisatellite mutations producing novel alleles increase the likelihood that distinctive combinations of *linked* alleles will be maintained, whereas the typical pattern of stepwise loss or gain of single repeat units at STRs usually reproduces existing alleles thereby diminishing such associations, assuming comparable mutation rates for the two types of loci (see below). There was no evidence of LD (P < 0.05) between any minisatellite and any STR which might lead to artefactual concordance.

### 3.4 MPS analysis of STRs

**Fig. 3:**
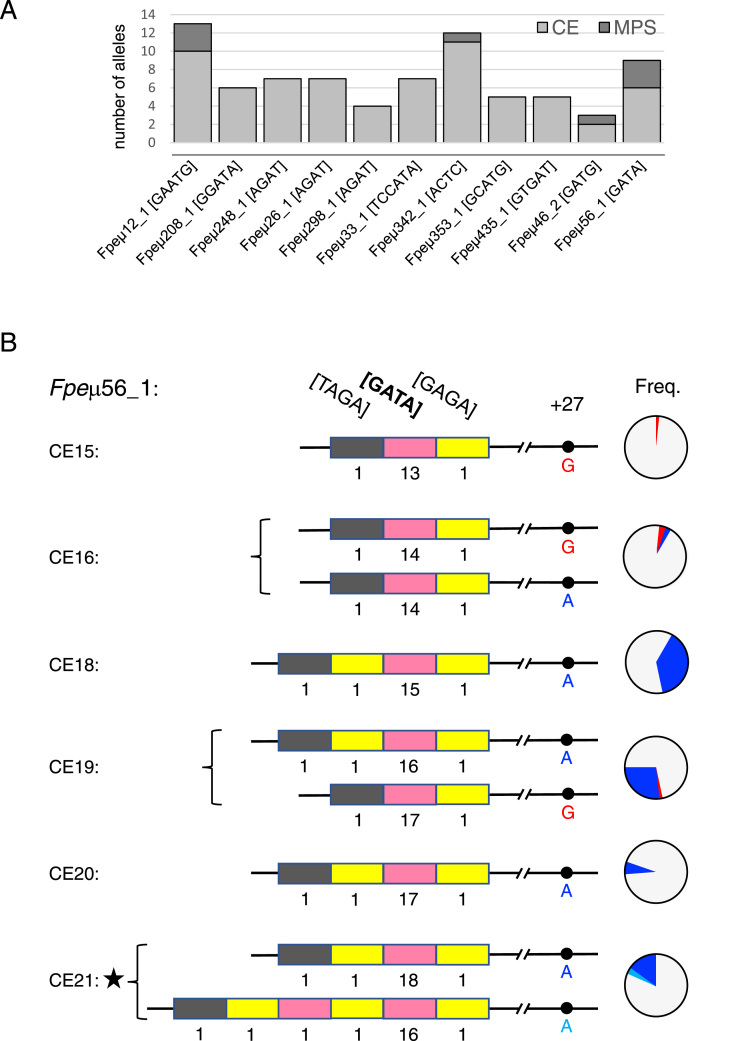
Additional variation revealed by MPS. (A) Graph showing the number of alleles detected by CE (pale grey bars) and MPS (dark grey bars) for each of the 11 STRs. (B) Schematic allele structures are shown for *Fpe*μ56_1 together with their CE lengths indicated on the left and their frequencies amongst the 31 unrelated birds on the right. Most variation is seen within a single GATA[n] repeat array (pink) (n = 13–18 copies), but additional tetranucleotide repeats upstream of this can also vary in sequence and/or number, as can the nucleotide at the 27th position downstream of the terminal GAGA repeat which can be either a G as in the published genome sequence (red), or an A (dark or light blue). Pie charts to the right indicate the frequencies of the different allele structures with the colour of the slice corresponding to the status of this flanking SNP. Two birds typed by CE as length homozygotes were identified by MPS as heterozygotes carrying isometric alleles (black star). (For interpretation of the references to colour in this figure legend, the reader is referred to the web version of this article.)

The 12 informative STRs identified bioinformatically from the published peregrine genome were amplified together in a single reaction for each of the 49 birds and then analysed in a single MPS run. The total number of paired sequence reads obtained per bird ranged from 3133 to 60,294 (mean 20,985). Allele calls from these sequences were made using FDSTools, open-source software specifically developed for forensic analysis of STR data ([56]). The minimum number of reads per STR locus per bird was 9 and the maximum 13,061, with an overall mean read depth of 1717 (Table S3). Read depth per locus exceeded 100 reads for all but a single STR in three different birds and with the exception of these there were at least 35 reads for each called allele. The MPS allele calls were found to be concordant with the CE data once differential band shifts caused by the attached fluorophores and sequence composition were taken into account ([16], see also Fig. S1 legend); the only exception was locus *Fpe*μ145_1 which showed considerable heterozygote imbalance with occasional loss of the larger allele and so was dropped from further analysis. The sequencing revealed additional allelic variation at four loci (*Fpe*μ12_1, *Fpe*μ46_2, *Fpe*μ56_1, *Fpe*μ342_1). In total a further eight alleles were observed with the distinguishing variation occurring both within the repeat arrays themselves and/or in the flanking DNA (Fig. 3, Table S4), as has been noted for some human forensic STR markers typed via MPS [17].

Although *Fpe*μ2 was not included in the MPS multiplex, Sanger sequencing was used to reveal the underlying cause of intermediate (0.5) alleles at this locus. In all cases examined this was due to the duplication of a CAAGA motif after the 3’ end of the hexamer repeat array, a motif that is also variable in the saker. In addition to the GGAAGA canonical repeats, the variant hexamer GGATGA was interspersed in some alleles, although the GGAAGG and GGAAA repeats noted in the atypically short peregrine reference genome allele were not observed in this study (see Table S5). Two examples of isometric alleles were found amongst the ten allele structures identified through sequencing, indicating the potential value of integration of this marker in future iterations of the MPS multiplex.

### 3.5 Power of discrimination

The ability of each marker set (minisatellite SLP, CE STR and MPS STR), to address issues of parentage and identity can be estimated from combined non-exclusion probabilities using the allele frequencies among the 31 unrelated birds (see Table 2). When only one claimed parent is available the probability of false inclusion is < 1% for the seven effectively unlinked minisatellites and < 2% for the co-amplifiable STRs, however when both adults are tested false inclusions are reduced to < 0.001% for all marker sets. The probability of false exclusion of a true parent or breeding pair is determined by the mutation rate, and in the case of STRs by the inheritance of undetected parental null alleles by seemingly homozygous offspring as well. The mean per-generation mutation rate at avian minisatellites, including those typed here, is estimated to fall in the range 0.01–0.001 [49] and a similar range could be conservatively applied to the STRs [4]. Whilst Fig. 1 shows shared alleles occur at every locus in a small proportion of pairwise comparisons between unrelated individuals (0.35% for minisatellites, 0.26% for STRs), from Fig. 2 we see that there were at least six loci with incompatible alleles within each marker set between excluded “offspring” and their claimed parents, and when randomised against all breeding pairs there were at least three loci per marker set, providing confidence that parentage could be accurately determined with either marker type. Comparison of the combined non-exclusion probability for identity also shows little difference in the power of the minisatellite and STR marker sets, demonstrating that the STR multiplexes are a suitable replacement for the previously validated and court-tested SLPs for determining a common origin for biological traces.

**Table 2:** Probabilities of false inclusion and identity with marker sets. Combined non-exclusion probabilities for first parent (NE-1P) where the second parent is unknown, parent pair (NE-PP), identity (NE-I) and sib identity (NE-SI) are presented for: all 22 markers, the eight autosomal minisatellites, seven minisatellites excluding *Fpe*MS17 due to strong LD with *Fpe*MS15, the 14 STRs typed by CE, the 12 bioinformatically selected STRs that amplify under the same multiplex conditions and the 11 STRs (excluding *Fpe*μ145_1) scored in the single MPS plex.

	All 22	8 Minis	7 Minis	14 STRs	12 STRs	11 STRs
NE-1P	1.03E-05	4.18E-03	7.71E-03	2.47E-03	1.33E-02	2.06E-02
NE-PP	1.18E-14	3.62E-07	1.91E-06	3.23E-08	2.64E-06	8.51E-06
NE-I	1.71E-24	3.99E-11	6.65E-10	4.50E-14	3.61E-11	3.32E-10
NE-SI	1.34E-09	2.23E-04	6.24E-04	6.01E-06	5.20E-05	1.27E-04

## 4 Discussion

The past thirty years have seen great advances in forensic genetics with clear applications to the investigation of wildlife crime, illustrated here by reanalysis of one the earliest cases with the first application of massively parallel sequencing of STRs for relationship testing in non-human vertebrates. Human DNA fingerprinting protocols which could be directly applied to animal DNA were rapidly replaced in the early 1990s by single-locus minisatellite profiling more suited to pairwise comparisons among large numbers of individuals. In 1993 the first SLP tests were carried out on birds of prey held in captivity. In that year, the BRS showed there were 154 goshawks and 360 peregrines declared as captive bred, though a small unknown proportion of the peregrines were hybrids (probably ≤ 60 birds). Though less than 20% of all declared 1993 progeny were tested, this work showed 39 peregrines (~13% allowing for hybrids) and 18 goshawks (11.6%) were not related to their declared parents, clearly indicating that significant numbers of birds declared as captive bred were actually taken from wild populations [36], [48]. There were a number of high-profile successful prosecutions as a result, with this case, and another large-scale peregrine laundering investigation, leading to custodial sentences. In 1994 the number of peregrine and goshawk offspring declared as captive-bred under the BRS fell by approximately 20%. This was suspected to be due to the deterrent impact of DNA profiling and the increased likelihood of detection and prosecution [13], [36]. Indeed, by 1996 SLP DNA profiling on samples from 69 registered goshawks and peregrines collected during announced inspections confirmed that all the offspring were bred from the claimed parent birds [13], [51]. Whilst the impact of SLP testing on theft from nests was significant, it was recognised that a more cost-effective method would be needed if a registration scheme were to be accompanied by routine DNA profiling.

An STR multiplexing system, mirroring that developed for human forensics, would be cheaper, faster, more sensitive, and straightforward for databasing. Initial attempts to apply such systems were limited by the STRs cloned and sequenced at the time [9], [30] which were predominantly dinucleotide repeats rather than the tetranucleotide repeat units favoured in human forensics and now also championed for non-human DNA forensic investigations [8], [22], [27], [42] as well as molecular ecology (e.g. [7]). Tetra-, and indeed penta- and hexanucleotide repeats show the ideal combination of characteristics - many alleles and high heterozygosity (resulting in high discrimination power), reasonably short total allele length (typically < 300 bp, allowing amplification from trace and degraded DNA) and clear amplification patterns (limited stutter and clear separation between alleles) in contrast to the profiles obtained from more abundant, and therefore more readily cloned, dinucleotide repeats [1]. In recent years, the need to select STRs from cloned libraries of fragmented DNA has diminished as they can now be identified directly by data-mining of genomic sequences that are becoming available. Screening genomes returns many more STRs than are required to provide robust forensic statistics and so a subset of loci which will amplify under similar conditions can be chosen and assayed together in a single multiplex. Constraints on multiplex size imposed by the limited number of fluorescent labels that could be used in CE to differentiate between loci with overlapping size ranges, are significantly eased by adopting the MPS approach.

Inadvertent selection of linked loci will also become rarer as improved sequencing approaches enables the many genome sequences that still comprise hundreds or thousands of short scaffolds to be linked into chromosome level assemblies [33]. STRs widely separated on the same chromosome may not necessarily be a concern as even quite small family groups can reveal evidence of frequent recombination between physically linked loci but examination of many, or larger, pedigrees is still desirable to determine whether closer associations might adversely affect forensic interpretation. Alternative, albeit less direct means of evaluating independence of markers used for wildlife crime investigations, such as determining population level associations via measures of LD have been proposed [47], though this requires adequate numbers of unrelated individuals all from the same and relevant population which may still be challenging to obtain. Defining the relevant population depends upon the case circumstances, and ideally both the captive and wild populations should be extensively sampled in order to establish the degree of differentiation. The 1990s UK captive peregrine population had been largely established from UK wild stock, including birds taken under licence from the wild (permitted until 1988), ongoing legal integration of wild disabled birds and a significant number of laundered wild birds. Further diversity came from smaller numbers of imported birds and limited hybridisation with other falcon species [13]. The use of individuals sampled during casework in this study provided a representative panel of unrelated individuals comprising birds drawn both from the wild populations being illegally exploited (largely Scottish in this instance) and the captive population into which they are being laundered.

For some forensic applications, such as testing the legitimacy of claimed parent/offspring relationships, even close linkage will not risk false prosecution as the absence of shared alleles across several loci is all that is needed to refute a false claim and this can only arise through multiple mutation events (with associated low probabilities), the inheritance of null alleles at several loci or misassignment of parentage. Questions of common origin are more seriously impacted due to the increased likelihood of sharing a suite of linked alleles. In this study the linked STR loci are all separated by at least 10.5 Mb, greater than the 6.3 Mb between vWA and D12S391, two loci in the standard European human DNA profiling multiplex, which are usually treated as unlinked except in cases (e.g. incest) where discrimination between very close kin is required [32]. While greater physical distance is likely to increase the likelihood of recombination the rate varies throughout the genome. For the two human loci the recombination fraction in multigeneration pedigrees was estimated as 0.108 [6]: from our very limited data we see a similar recombination fraction of 0.083 or more between all but one pair of loci and so in combination with the absence of detectable LD among unrelated birds an assumption of independence is reasonable if the likelihood of close relationship is low. As seen within the legitimate sibling group in this case physical linkage can result in anomalously high or low estimates of the coefficient of relatedness but this will diminish with each generation as recombination breaks up linkage groups. To account for the potential persistence of association between alleles among birds who are believed to be unrelated, the use of extremely conservative measures of the probability of identity based on the probability of siblings sharing identical profiles has been proposed [45]. In this case our marker sets still yield combined probabilities of non-exclusion of sib identity (NE-SI) of < 0.001.

The STR markers described here offer similar discrimination power to the minisatellite loci that were used in the 1990s but with greater ease of analysis and reduced sample requirements (~30 ng, fragment length 100–500 bp). Although CE analysis of the multiplexes required several independent amplifications to split loci with similar size distributions into distinguishable sets, the MPS approach, for which they were ultimately intended, allows them to be combined in a single amplification and demultiplexed bioinformatically into individual loci whilst keeping amplicon lengths as short as possible. Furthermore, we have demonstrated here that additional discrimination amongst predominantly UK-derived peregrines can be gained using MPS, either by using loci with compound repeat structures and/or including polymorphism information in the flanking DNA. The MPS approach is likely to be most suited to complex persecution casework where limited amounts of degraded and/or mixed DNA may have to be analysed to link a suspect or item to a shot, trapped or poisoned bird. This has increasingly been the focus of UK raptor investigations [35].

The nature of raptor crime within the UK has also changed in other ways over the intervening period following a shift towards the production of hybrid falcons by artificial insemination that accelerated dramatically from the mid-1990s as the take from the wild in the UK declined. Prior to 1994, hybrid falcons had made up a negligible proportion of UK captive breeding claims but by 2003 they represented more than 70% of submissions, leading to growing concern about their escape and potential genetic introgression into the wild population [13]. This has led to an additional requirement of distinguishing between pure and hybrid birds. The STRs described in this study were chosen with this in mind and were tested across a range of falcon species to ensure that they were polymorphic in all. Initial data suggest that whilst the allele size ranges overlap between species the additional information in the repeat structures and flanking polymorphisms detectable by MPS may prove useful in determining a bird’s origins. Furthermore, these markers, and the parallel set developed for accipiters using similar approaches and selection criteria [2], could be applied globally in the study of wild populations to explore interaction between subpopulations and sympatric species, and recovery following population bottlenecks [5], [14], [21], [40], [52].

The application of SLP profiling to tackle the illegal laundering of birds of prey during the 1990s is widely regarded as one of the most successful examples of the use of a forensic technique to investigate UK wildlife crime. It also led to new legislation being introduced to facilitate the collection of samples to undertake identity or ancestry checks. The success of DNA profiling was dependent on three key aspects of the BRS; birds were individually and uniquely identifiable by numbered leg-rings or microchips, the physical location of individual birds was known, and information on all the declared familial relationships was available. Unfortunately, in 2008 (2009 in Scotland and Wales) the government reduced registration controls on peregrines kept in captivity in Great Britain. This effectively made it substantially more difficult for the statutory agencies to locate birds suspected of being taken from the wild once they had been sold or moved on by the breeder. Without this audit trail the value of DNA profiling in enforcement has been severely hampered. Concerningly, the last two decades have seen a significant rise in the sport of falcon racing in the Middle East which has again created an increased demand for peregrines and other falcons. Unsurprisingly, this has led to a substantial increase in UK prices, with female peregrines fetching £5000 or more.

Around 2008, about 350 peregrines were declared captive bred each year, about the same number as the 1990s. Some ten years on this had more than doubled, and hundreds of these birds were being exported outside the EU. In the UK, eggs and chicks continue to be taken from some wild peregrine nests, so there seems little doubt that an unknown percentage of birds declared as captive bred are in reality illegally taken from the wild. Consequently, there is a need for DNA testing on captive breeding claims of those suspected of illegally dealing in wild taken birds, in conjunction with an annual programme of random checks, akin to the work undertaken in 1996. This work needs to be focussed on the period shortly after birds are hatched and before they are sold or moved on. This approach would provide a strong deterrent for those illegally dealing in falcons and allow some assessment of the scale of the problem. Having validated, highly discriminating and cost-effective DNA profiling methods to assess the legitimacy of captive breeding claims will be a vital component of any such work and the STR markers reported for the first time here will provide the basis of such tests.

## Conflicts of interest

None.
