# Supplementary material for: Massively parallel sequencing and capillary electrophoresis of a novel panel of falcon STRs: Concordance with minisatellite DNA profiles from historical wildlife crime
Source: Forensic Sci Int Genet. 2021 Sep;54:None. doi: 10.1016/j.fsigen.2021.102550 (PMC8430417; doi:10.1016/j.fsigen.2021.102550)
Supplement: Supplementary file 1 — Supplementary material [file mmc1.pdf]

Table S1: Primer details for the CE multiplexes

CE Plex	STR<br>[major repeat motif]	Fluor	F primer<br>(5’ to 3’)	R primer<br>(5’ to 3’)	Conc’n μM
FAL1	*Fpe*μ298_1<br>[AGAT]	6-FAM	CCCCAAGCATCTTTCTGTGG	CCGATGCACAAGGTTCACAA	0.17
	*Fpe*μ46_2<br>[GATG]	VIC	GGTGGATGAGTATTGGCTTCC	GGCCACCACGTATGTTTTGA	0.12
	*Fpe*μ342_1<br>[ATCT]	NED	CCCATCCTGTCCAAATGCAG	GGCGTTTCCTGGAGATAAGAG	0.12
	*Fpe*μ435_1<br>[GTGAT]	PET	GGCAGTTTTCACACCTGGTT	GACAAGACATGGAGCAAGCA	0.45
FAL2	*Fpe*μ208_1<br>[GGATA]	6-FAM	TGACTCTGGCAGCGTATCAG	TCCTTGTCCTTTGGCTGTCT	0.18
	*Fpe*μ248_1<br>[AGAT]	NED	CAGCATGTTTTGGCCTGGAT	GGTTGACCGGGAAGTAAAGTG	0.06
	*Fpe*μ26_1<br>[AGAT]	PET	CTGTTGCACTGGATCTAGCC	CCCCGCAAAACACAAGTGAT	0.27
	*Fpe*μ25_1<br>[CTATC]	VIC	GGAGGGATTTGGACAACACC	AGGTCCAGGTCATAATGAAGGT	0.14
FAL3	*Fpe*μ145_1<br>[CCCAT]	6-FAM	TAGGGCGGTATAACTGTGCC	GAAACAAAAGTCAGGTTGCCA	0.17
	*Fpe*μ353_1<br>[GCATG]	NED	CACGTAGCAGCAGTTGGATC	TGGAAGATGCCTGTGGAAAA	0.37
	*Fpe*μ98_2<br>[AGAT]	PET	AGCCAGCAGACATATTAGCCA	ATCTCTGGGTGAACTGGGTG	0.56
	*Fpe*μ12_1<br>[GAATG]	PET	GTGCACCCCAAAGCTACTTC	GCATTGCATCTATTCTGTTCCG	0.08
FAL4	*Fpe*μ33_1<br>[TCCTA]	VIC	CGTGTGTGAGAGGCATTTGG	GCTACAGGAGGAGGTGTACC	0.2
FAL5	*Fpe*μ56_1<br>[GATA]	VIC	TTGTGTAGGCAAGGCTAGGG	TTTGCAACACTACGTCCACC	0.2
FAL6	*Fpe*μ1<br>[GATG]	NED	TGTAAGTGGTGTTAAAACAG	GATATTAATTCCAAAGTCCA	0.2
	*Fpe*μ2<br>[GGAAGA]	PET	GGTCATTAACGCATGCATCC	TCGTAGGATGCTTCTAACTG	0.2

Table S2: Locations of the STR markers

STR	Scaffold accession [size, bp]	Position in scaffold: start, bp	Position in scaffold: end, bp	Scaf str'd	PCF ID	Ori PCF	Chr	Chromosome accession	Distance between STRs, Mb	Number of pedigree recomb'nts, **
*Fpe*μ12_1	NW_004930387.1 [4,101,875]	3,442,740	3,442,957	+	4b-b	Y	2	CM007506.1	NA	NA
*Fpe*μ248_1	NW_004930217.1 [1,595,755]	129,395	129,609	+	2h_2d	Y	3	CM007507.1	NA	NA
*Fpe*μ33_1	NW_004930110.1 [4,471,847]	1,432,208	1,432,448	+	1f	N	4	CM007508.1		
*Fpe*μ2 (*Fpe*μ127_2)	NW_004930405.1 [2,723,483]	1,987,640	1,987,812	+	1f	N			10.5	1
*Fpe*μ56_1	NW_004929840.1 [6,659,497]	3,853,892	3,854,132	+	1a_30a	Y			16.1	0
*Fpe*μ1 (*Fpe*μ9_1)	NW_004929449.1 [11,051,831]	4,519,254	4,519,428	-	1b_30b	Y			19.0	2
*Fpe*μ25_1	NW_004930203.1 [3,995,603]	3,172,019	3,172,266	+	1b_30b	Y			7.7	NA
*Fpe*μ298_1	NW_004930159.1 [401,964]	295,610	295,810	+	1b_30b	Y			6.9	1
*Fpe*μ98_2	NW_004929458.1 [6,943,041]	6,922,723	6,923,101	+	2a	Y	5	CM007509.1	23	NA
*Fpe*μ145_1	NW_004930347.1 [510,521]	59,388	59,586	+	2a	Y				
*Fpe*μ208_1	NW_004930269.1 [6,137,192]	5,692,833	5,693,054	+	29b	Y	6	CM007510.1	NA	NA
*Fpe*μ26_1	NW_004930193.1 [18,327,016]	15,209,789	15,209,944	+	3e	Y	7	CM007511.1	NA	NA
*Fpe*μ435_1	NW_004929998.1 [388,920]	21,102	21,343	+	33c	U	Z*	NA	NA	NA
*Fpe*μ342_1	NW_004930107.1 [1,854,119]	210,016	210,259	+	2b	U	NA	NA	NA	NA
*Fpe*μ353_1	NW_004930096.1 [2,490,089]	1,974,775	1,974,971	+	2e	U	NA	NA	NA	NA
*Fpe*μ46_2	NW_004929952.1 [432,680]	292,104	292,351	+	?	NA	NA	NA	NA	NA

Scaf str'd = orientation of STR within scaffold

PCF = predicted chromosome fragments determined by Damas *et al.* 2017

Ori PCF = orientation of PCF in chromosomal assembly established by Damas *et al.* 2017; Y = yes, N = No, U = unplaced scaffold

* = as determined by inheritance pattern in this study

? = not determined by Damas *et al.* 2017

** relative order of *Fpe*μ33_1, *Fpe*μ2 and *Fpe*μ56_1 established in this study from the number of recombinants noted amongst legitimate families.

NA = not applicable

Table S3: MPS read depth by locus

STR	min reads per bird	max reads per bird	mean reads per bird	min reads per called allele	
Fpe µ12_1	28	5,885	1,781	19	next lowest 113
Fpe µ208_1	43	5,171	2,145	23	next lowest 157
Fpe µ248_1	9	6,282	1,640	9	next lowest 151
Fpe µ26_1	479	13,061	3,852	300	
Fpe µ298_1	416	4,962	2,303	323	
Fpe µ33_1	197	4,548	1,447	67	
Fpe µ342_1	263	4,398	1,534	109	
Fpe µ353_1	202	4,847	1,743	163	
Fpe µ435_1	84	2,487	765	49	
Fpe µ46_2	191	3,163	1,113	63	
Fpe µ56_1	124	1,977	721	41	
Fpe µ145_1	119	4,390	1,559	35	
	9			overall min number of reads per locus per bird	
		13,061		overall max	
			1,717	overall mean read depth per locus	

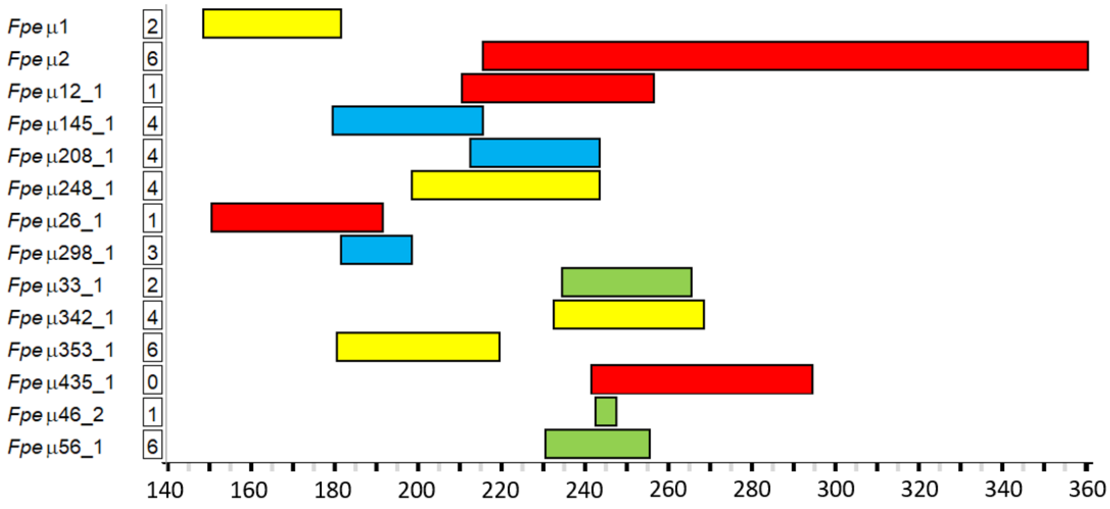

Figure S1: Observed size ranges of the 14 STRs assayed by CE

The observed allele size ranges (in bp) determined by CE are shown for each of the 14 STRs. It was noted that these differ from the expected base pair lengths according to the genome reference and MPS data (Table S3) in a manner most likely dependent upon both the sequence and fluorophore (6-FAM=blue, NED=yellow, VIC=green and PET=red); the numbers within each box to the left indicate how much smaller (in bp) the CE sizing appeared. Hahn *et al.* (2001) reported similar effects for amplicons labelled with 6-FAM and NED. The use of allelic ladders for sizing alleles would mitigate this effect.

Supplementary text

Single Locus Profiling

After initial multilocus profiling (MLP) with the red kite (*Milvus milvus*) probe c*Mmi*30 (May *et al.* 1993) which suggested multiple incompatibilities with registration documents, single-locus profiling (SLP) followed the methods of Wetton *et al.* (1995) using probes derived from charomid clones isolated from several species: c*Mmi*12 isolated from the red kite which detects a polymorphic W chromosome linked band in peregrines (May 1993, Wetton & Parkin 1994), c*Fpe*MS1, c*Fpe*MS5, c*Fpe*MS13, c*Fpe*MS15 and c*Fpe*MS17 all from the peregrine (*Falco peregrinus*), c*Fco*MS2 and c*Fco*MS19 from the merlin (*Falco columbarius*) and c*Fti*1 from the kestrel (*Falco tinnunculus*) (Wetton & Parkin 1997). 1.5-μg aliquots of *Mbo*I-digested peregrine DNA along with 10 ng of an internal lane standard comprising a 4:1 mix of 1 kb ladder and *Xho*I digested lambda DNA were loaded onto 30-well, 22 cm 0.8% (w/v) agarose gels and electrophoresed for 42 hrs at 42 V before Southern blotting onto ZetaProbe GT nylon membranes. ^{32}P-labelled probes were hybridized overnight followed by low stringency washes at 1xSSC (MLP and SLP) and an additional high stringency wash at 0.1xSSC (SLP only) to reduce non-specific binding of the probes, then overnight autoradiography with two intensifying screens with additional longer exposures as appropriate (typically 3-5 days) to reveal the appropriate locus. Once scored each blot was stripped of probes by addition of boiling 0.1% (w/v) SDS prior to successive re-hybridizations with up to four other probes. Autoradiographs were scored by eye and alleles assigned sequential alphabetic codes where they could be confidently distinguished by reference to samples repeated both across and between the blots, and the internal lane standard. As minisatellites typically have a repeat unit length of 10-60 bp and a repeat array length of 1-16 kb it is not possible to precisely determine the size of alleles and so alphabetic codes act as “bins" that may include more than one allele length. Full details of the development of the SLP tests from cloning the single locus probes to analysis of autoradiographs can be found in Wetton & Parkin 1994.

Isolation of STRs by cloning

Markers *Fpe*μ1 and *Fpe*μ2 were identified from a library of cloned tetramer-enriched peregrine DNA using the methods of Armour *et al.* (1994) (Neumann & Wetton 1996). Primer sequences were designed by eye following Sanger sequencing of isolated plasmid DNA.

References (Supplementary text only)

Armour J.A., Neumann R., Gobert S., Jeffreys A.J., Isolation of human simple repeat loci by hybridization selection Hum. Mol. Genet. 1994 599-605

May C.A., Wetton J.H., Parkin D.T., Polymorphic sex-specific sequences in birds of prey

Proc. R. Soc. Lond. Ser. B Biol. Sci. 1993 271-276

Neumann K., Wetton J.H., Highly polymorphic microsatellites in the house sparrow Passer domesticus Mol. Ecol. 1996 307-309

Wetton J.H., Burke T., Parkin D.T., Cairns E., Single-locus DNA fingerprinting reveals that male reproductive success increases with age through extra-pair paternity in the house sparrow (Passer domesticus) Proc. R. Soc. Lond. Ser. B: Biol. Sci. 1995 91-98
